# Supplementary material for: The effects of vertical trunk supportability improvement on one-leg rebound jump efficiency
Source: PLoS One. 2022 May 19;17(5):e0267460. doi: 10.1371/journal.pone.0267460 (PMC9119522; doi:10.1371/journal.pone.0267460)
Supplement: Attachment — Submitted filename: Response to Reviewers.docx [file pone.0267460.s001.docx]

The effects of vertical trunk supportability improvement on one-leg rebound jump efficiency

The changes in response to the academic editor

The author sincerely appreciates the opportunity to improve the manuscript that has been provided by the academic editor. Please find below the improvement and an explanation given by the author based on the academic editor’s comments and recommendations.

| 1. Please ensure that your manuscript meets PLOS ONE's style requirements, including those for file naming. | Thank you for your comment.  (Line 21, 23-24) |
| --- | --- |
| 2. We note that you have indicated that data from this study are available upon request. PLOS only allows data to be available upon request if there are legal or ethical restrictions on sharing data publicly. For more information on unacceptable data access restrictions, please see http://journals.plos.org/plosone/s/data-availability#loc-unacceptable-data-access-restrictions.  In your revised cover letter, please address the following prompts: | Thank you for your comment.  This data is related to privacy such as personal information. It is not possible to share data from third parties freely. In such cases, the author will make the data available upon request.  The ethics committee of Shijonawate Gakuen University  Department of Physical Therapy, Faculty of Rehabilitation, Shijonawate Gakuen University, Osaka 574-0011, Japan |
| 3. PLOS requires an ORCID iD for the corresponding author in Editorial Manager on papers submitted after December 6th, 2016. Please ensure that you have an ORCID iD and that it is validated in Editorial Manager. To do this, go to ‘Update my Information’ (in the upper left-hand corner of the main menu), and click on the Fetch/Validate link next to the ORCID field. This will take you to the ORCID site and allow you to create a new iD or authenticate a pre-existing iD in Editorial Manager. Please see the following video for instructions on linking an ORCID iD to your Editorial Manager account: https://www.youtube.com/watch?v=_xcclfuvtxQ" | Thank you for your comment.  I have registered for ORCID iD.  The registration number is below.  [https://orcid.org/0000-0001-6140-3178] |
| 4. Thank you for submitting the above manuscript to PLOS ONE. During our internal evaluation of the manuscript, we found significant text overlap between your submission and the following previously published works.  ①https://www.jstage.jst.go.jp/article/jpts/31/3/31_jpts-2018-388/_pdf/-char/en  ②https://www.frontiersin.org/articles/10.3389/fphys.2019.01462/full  ③https://www.jospt.org/doi/pdfplus/10.2519/jospt.2007.2331 | Thank you for pointing this out.  Corrections have been made in the relevant sections.  ①Line 76-77  ②Line 246-248  ③ Line 267-269 |

The effects of vertical trunk supportability improvement on one-leg rebound jump efficiency

The changes in response to the reviewer # 1

The author sincerely appreciates the opportunity to improve the manuscript that has been provided by the reviewer.

Please find below the improvement and an explanation given by the author based on the academic reviewer’s comments and recommendations.

* Corrected sections are highlighted in the text (yellow).

| The duration of trunk training is not clear. | Thank you for your comment.  The text has been added and revised.  (Line 187-192, L194) |
| --- | --- |
| The characteristics of participants are missing. | Thank you for your comment.  The text has been added and revised.  (Line 94, Line97-98) |
| Statistical analysis is not explained. | Thank you for your comment.  The text has been added and revised.  (Line 204-209) |
| The stabilometry measure is not well explained, Why the authors do not used a stabilometric force platform? | Thank you for your comment.  Use the force platform.  (Line 146, 156, Figure 2 has been added.) |
| In my opinion in the discussion should be more references, especially in this affirmation "Therefore, jump efficiency is important for activities. These results suggest that vertical trunk supportability is important in bipedal animal activity and is useful in longitudinal studies. The strength of the lower limbs can be considered to improve jump efficiency. The results of this study showed that the maximal isometric knee extensor strength test of the lower limb strength index increased significantly from pre-to post-intervention. However, there was no significant change from post-detraining. Therefore, this result is not conside 195 red a mere increase or decrease in lower limb strength". In this affirmation ther is not refence "Lower limb strength results were also unaffected. The rebound jump has a greater centrifugal force on the trunk than on the lower limbs. The center of gravity of the trunk may lead to the next activity by being controlled quickly on the lower limbs. In other words, it is inferred that improvement of core function becomes easier to control to the next activity by changes in neuromuscular control or movement pattern" is necessary more references in the discussion there few and most are very old. | We agree with this concern shared by the reviewer.  The text has been added and revised.  (Line 254-263)  (Line 264-269)  (Line 271-278) |

The effects of vertical trunk supportability improvement on one-leg rebound jump efficiency

The changes in response to reviewer # 2

The author sincerely appreciates the opportunity to improve the manuscript that has been provided by the reviewer.

Please find below the improvement and an explanation given by the author based on the academic reviewer’s comments and recommendations.

* Corrected sections are highlighted in the text (yellow).

| Line 37-43: Please add more specific explanations on trunk function of anatomical (muscles) and biomechanical perspectives during movement.  Sentences within the first paragraph are vague and not informative, and further the authors did not provide sufficient data for a rational of this study. | We agree with this concern shared by the reviewer. The text has been added .  (Line 42-52)  Thank you for your comment. The text has been added and revised.  (Line 53, 57- 65) |
| --- | --- |
| Line 44-55: The authors mainly described a trunk righting test in the second paragraph, however, the authors did not explain how this test’s outcome can contribute to results of other tests, and how the trunk tithing test is associated other functional tests and further activities of daily living. It seems the authors did not provide thorough literature review in this area, and they need to add explanations why this study is important (a rational of this study)? | We totally agree with this concern from the reviewer.  The text has been added and revised.  (Line 69-77) |
| Line 44-55: No explanation on the intervention dose (a duration, frequency, set/reps, types, etc.) from previous studies. Thorough literature review is required, and should provide a sentence how this study bridges a gap from a current literature. | Thank you for your comment. The text has been added and revised.  (L187- 194) |
| Line 37: what activities? | Thanks for the comment. The text has been added and revised. (Line 53) |
| Line 41: what injury, what sports, what age? How can core stability be defined in that study? | Thank you for your comment. The text structure and content have been revised. (Line 53-67) |
| Line 57-151: Authors should provide a validity and reliability scores of all testing. They should add whether testing protocols were adapted from previous studies or not. If not, please add more pictures regarding testing procedures to better understand how the procedures are done. | We agree with this concern shared by the reviewer. The text has been added and revised. (Line 117-118, 129-131, 135-137, 141-143, 153-154, 164-166) |
| Line 166: As authors mentioned in the limitations of the study, not having a control group in the intervention study was a major concern. We can’t conclude whether pre-post changes were due to the intervention or other confounding factors. | We agree with this concern shared by the reviewer. This concern has been highlighted in the study limitations and is as follows. (Line 291-292) |
| Line 169-217: In the discussion section authors should add more relevant citations for each sentence. Some sentences are required supporting evidence, but it’s missing. | We agree with this concern shared by the reviewer. The text has been added and revised.(Line 246-248, 254-263, 264-269, 271-278, 282-288) |
