# Supplementary material for: The effects of vertical trunk supportability improvement on one-leg rebound jump efficiency
Source: PLoS One. 2022 May 19;17(5):e0267460. doi: 10.1371/journal.pone.0267460 (PMC9119522; doi:10.1371/journal.pone.0267460)
Supplement: Attachment — Submitted filename: Response to Reviewers.docx [file pone.0267460.s002.docx]

The effects of vertical trunk supportability improvement on one-leg rebound jump efficiency

The changes in response to the academic editor

The author sincerely appreciates the opportunity to improve the manuscript that has been provided by the academic editor. Please find below the improvement and an explanation given by the author based on the academic editor’s comments and recommendations.

| 1. Please review your reference list to ensure that it is complete and correct. If you have cited papers that have been retracted, please include the rationale for doing so in the manuscript text, or remove these references and replace them with relevant current references. Any changes to the reference list should be mentioned in the rebuttal letter that accompanies your revised manuscript. If you need to cite a retracted article, indicate the article’s retracted status in the References list and also include a citation and full reference for the retraction notice. | Thank you for your comment.  Previous manuscript：  17) Kisner C, Colby LA. Therapeutic Exercise. Foundations and Techniques. Philadelphia, PA: F. A. Davis Company; 1990.  　　　　　　　　　↓ change  The current manuscript：  29) Cabrejas C, Solana-Tramunt M, Morales J, Campos-Rius J, Ortegón A, Nieto-Guisado A, et al. The Effect of Eight-Week Functional Core Training on Core Stability in Young Rhythmic Gymnasts: A Randomized Clinical Trial. Int J Environ Res Public Health. 2022; 19: 3509.  I have changed my references. The reason is that the previous manuscript was an old textbook, so I changed it to a more recent paper. |
| --- | --- |

The effects of vertical trunk supportability improvement on one-leg rebound jump efficiency

The changes in response to the reviewer # 1

The author sincerely appreciates the opportunity to improve the manuscript that has been provided by the reviewer.

Please find below the improvement and an explanation given by the author based on the academic reviewer’s comments and recommendations.

* Corrected sections are highlighted in the text (yellow).

| Line 42 In this phrase "Trunk stability involves many muscles, and these muscles are classified into local and  43 global muscles [32]" it should be reference [1]. References should be listed in numerical order, and in the same order in which they are cited in text per example. 1,2,3,4,etc | Thank you for your comment.  I have changed the list of references in the manuscript. |
| --- | --- |
